# Reading and Language Disorders: The Importance of Both Quantity and Quality

**DOI:** 10.3390/genes5020285

**Published:** 2014-04-04

**Authors:** Dianne F. Newbury, Anthony P. Monaco, Silvia Paracchini

**Affiliations:** 1 Wellcome Trust Centre for Human Genetics, University of Oxford, Oxford OX3 7BN, UK; 2 Tufts University, Ballou Hall, Medford, MA 02155, USA; E-Mail: Anthony.Monaco@tufts.edu; 3 School of Medicine, University of St. Andrews, St. Andrews, KY16 9TF, UK; E-Mail: sp58@st-andrews.ac.uk

**Keywords:** Specific Language Impairment (SLI), dyslexia, genetics

## Abstract

Reading and language disorders are common childhood conditions that often co-occur with each other and with other neurodevelopmental impairments. There is strong evidence that disorders, such as dyslexia and Specific Language Impairment (SLI), have a genetic basis, but we expect the contributing genetic factors to be complex in nature. To date, only a few genes have been implicated in these traits. Their functional characterization has provided novel insight into the biology of neurodevelopmental disorders. However, the lack of biological markers and clear diagnostic criteria have prevented the collection of the large sample sizes required for well-powered genome-wide screens. One of the main challenges of the field will be to combine careful clinical assessment with high throughput genetic technologies within multidisciplinary collaborations.

## 1. Introduction

The disturbance of speech and language development is a common feature of many neurodevelopmental disorders [1]. Language impairment is often secondary to more pressing clinical features (e.g., in autistic disorders, epilepsy or periventricular heterotopia), but in some cases may represent the primary clinical concern (“specific language disorders”, e.g., in Specific Language Impairment (SLI) and dyslexia) [1]. Specific language disorders typically occur in the absence of any gross neurodevelopmental difficulties, neurological or sensory impairments and with normal non-verbal intelligence. SLI is defined as a disturbance of oral language skills, whereas dyslexia is an impairment in the use and/or understanding of written language [2]. Both show a strong familial bias, and heritability estimates indicate that a high proportion of the phenotypic variation in each of these disorders can be attributed to genetic variation [3,4,5].

## 2. Complex Traits, Complex Definitions

Whilst the terms “dyslexia” and “SLI” are widely used in the clinical and research literature, both disorders lack clear diagnostic guidelines and are often defined chiefly in terms of exclusionary criteria [6]. DSM-5 (Diagnostic and Statistical Manual) classifies SLI as a language disorder, while dyslexia is categorized as a specific learning disorder. However, both diagnoses require that “the individual’s difficulties must not be better explained by developmental, neurological, sensory (vision or hearing), or motor disorders and must significantly interfere with academic achievement, occupational performance, or activities of daily living” [7]. To complicate things further, deficits of language may vary considerably both between individuals and over the developmental trajectory in addition to the modality of language affected [6,8]. It is generally accepted that dyslexia primarily reflects a difficulty in the domain of phonological decoding (translating written letters into speech sounds) [9]. Nonetheless, it is very common to observe a phonological deficit in combination with other manifestations, like sensory or fine motor control problems [10,11]. SLI has been proposed to reflect a deficit in phonological short-term memory (the retention of verbal information for short periods of time) [12], auditory perception (the processing of brief and/or rapid auditory stimuli) [13] and/or the development and application of grammatical rules [14]. However, all of these theories are supported by varying amounts of evidence in the primary research literature.

## 3. Changing and Heterogeneous Phenotypes

In addition, the difficulties experienced by any given individual may cross linguistic and cognitive domains and often change as the child develops [15,16]. Such observations perhaps suggest that SLI and dyslexia cannot be treated as discrete clinical conditions. Instead, it is possible that these language disorders might represent complex end effects of the disruption of multiple cognitive development processes that overlap with, and are related to, the secondary language disorders mentioned above [17]. Under this model, the investigation of dimensions of impairment may be more relevant than the ascertainment of clinical cohorts. The observed co-occurrence of SLI and dyslexia (~50%, [18]) support such a hypothesis and has led researchers to suggest that both disorders may result from an impairment in phonological representation [17]. The clinical presentation of the deficit may represent the severity of the underlying impairment or the presence of additional language- or cognitive-related difficulties [17,19]. Thus, “specific” language disorders may be the exception, rather than the rule, since co-morbidities often extend outside of the linguistic domains. For example, weaknesses in motor skills and executive function and reduced functional brain laterality are commonly described in children with dyslexia or SLI [20,21,22,23,24,25,26]. Nonetheless, the causal relationships between symptoms and cognitive, linguistic and developmental markers have yet to be elucidated. Others maintain that SLI and dyslexia may still have separate etiologies, and the co-incidence may simply represent comorbidity, as seen with other neurodevelopmental disorders [19]. Identifying the genetic underpinning of these disorders is required to inform this debate and reach more definitive conclusions about the diagnosis. For example, are there shared or partially overlapping genetic factors that contribute to separate disorders? Can we talk about common etiologies? Or do the DSM-5 definitions correlate with distinct biological pathways? While it would be tempting to simplistically ask if candidate genes for a single disorder can also influence a disorder with a different diagnosis, we might be asking the wrong questions if the initial diagnoses are artificial clinical constructs and misleading with regards to etiology.

## 4. Monogenic Conditions Back in the Picture

For many language disorders that are associated with known syndromes, the genetic cause of the syndrome itself is known. These involve a wide range of genetic mutations, from point mutations (for example, *MECP2* mutations in Rett syndrome [27]), to nucleotide expansions (for example, the expansion of the *FMR1* gene in Fragile X [16]) and deletion syndromes, such as velo-cardio-facial syndrome, which results from a 3-Mb deletion on chromosome 22q11.2 [28], or the duplication or deletion of an entire chromosome, such as Down syndrome and Turner syndrome [29,30,31]. Thus, it is likely that any population selected to have language impairment will harbor a subset of children with these recognized syndromes [32]. Single gene mutations have also been described for some specific language disorders. Mutations and disruptions of the *FOXP2* gene lead to childhood apraxia of speech [33,34], and point mutations in genes in the lysosomal pathway (*GNPTAB*, *GNPTG* and *NAGPA*) have been associated with persistent stuttering [35]. However, even in these severe and exceptional cases, there is often a high degree of heterogeneity between individuals. While some generalizations can be made, there is still considerable inter-individual variation. For example, individuals with *FOXP2* disruptions invariably present with dyspraxic speech (the inability to make fine-tuned oromotor movements necessary for coherent speech), while others can present with both receptive and expressive language difficulties, only an expressive deficit, only with intellectual deficits or good performance on non-verbal intelligence tasks [36].

## 5. Genetic Windows into Development

One argument against the utility of understanding the genetic underpinnings of these rare syndromic language disorders has been to question their relevance to the biology of common forms of SLI and dyslexia. However, the identification of a specific candidate gene and mutations thereof can allow the development of targeted investigations in cellular or animal models, which, in turn, can point to mechanisms that might be relevant to more common forms of language-related conditions affecting thousands of children. An example of this is how the FOXP2 transcription factor regulates the expression of target genes, such as *CNTNAP2*, which has been shown to play a role in more common forms of language impairment [37], as well as other neurodevelopmental disorders [38,39,40,41] and normal language variation [42]. The increased resolution and power of genetic screening demonstrates that the boundary between monogenic and common traits may actually be less defined than that predicted previously. Recent large-scale studies have clearly shown that genes disrupted by highly penetrant mutations and leading to well-defined diseases can play a role in complex disorders, although this may only be relevant in a subset of cases [43]. Nonetheless, genetic contributions to the majority of specific language disorders are expected to be complex in nature and involve genetic variation in many genes, which combine to determine an overall risk of disorder [44].

## 6. Classical Approaches

Genetic contributions to neurodevelopmental disorders (both syndromic and specific forms) can be traced by linkage analyses. This approach can be applied to extended multi-generational families or large collections of small nuclear families and can involve the investigation of linkage-disequilibrium patterns [45]. Linkage analysis allows the identification of broad chromosome regions that co-segregate with a disorder in a given family unit. Since such studies consider segregation patterns rather than specific genetic variants, they can enable the identification of shared chromosome regions even when the pathological variants in each region differ between family units. This methodology is particularly powerful for the identification of genetic variants with high penetrance and expressivity and can be applied to more complex situations, such as that expected for both SLI and dyslexia. 

## 7. SLI and Dyslexia Linkage Loci

Genome-wide screens or targeted analyses of dyslexic families have allowed the identification of linkage loci on chromosomes 15q21 (DYX1—OMIM#127700) [46,47], 6p22.3-p21.3 (DYX2—OMIM#600202) [46,48,49,50,51], 2p16-p15 (DYX3—OMIM#604254) [52,53], 3p12-q13 (DYX5—OMIM#606896) [54,55], 18p11.2 (DYX6—OMIM#606616) [56], 11 (DYX7—OMIM#127700) [57], 1p36-p34 (DYX8—OMIM#608995) [58,59] and Xq27.2-q28 (DYX9—OMIM#300509) [60]. Subsequent fine-mapping efforts across linked loci, through the investigation of specific genetic variations or the characterization of individuals with chromosome imbalances, have led to the identification of putative risk variants in the *DCDC2* [61], *KIAA0319* [62,63,64], *DYX1C1* [65,66], *C2orf3*/*MRPL19* [67], *CYP19A1* [68] and *ROBO1* [54] genes. Each of these candidate genes has a variable amount of support, ranging from observations limited to a single family to replication across multiple cohorts [69]. Nonetheless, functional analyses have led to an intriguing conversion upon pathways involving neuronal migration [70,71,72,73,74,75,76] and cilia motility [72,77,78,79], as discussed in later sections. Investigations of SLI are less advanced, but linkage studies have identified four loci of interest on chromosomes 7q35-q36 (SLI4—OMIM#612514) [80], 13q21 (SLI3—OMIM#607134) [81,82], 16q (SLI1—OMIM#606711) [83,84,85,86] and 19q (SLI2—OMIM#606712) [83,84,85,86], and subsequent studies have identified two candidate genes; *CMIP* and *ATP2C2*, both in SLI1 [87]. Microdeletions in the *ZNF277* gene on chromosome 7 have also been implicated in SLI [88], as has the Human Leukocyte Antigen (HLA) locus [89].

Although the linkage loci described for SLI and dyslexia do not overlap, studies of other complex genetic disorders indicate that there may be hundreds of genetic variants contributing to any one phenotypic status. Since genetic analyses are likely to detect only the major gene effects within any given cohort (the so-called winners curse [90]), this observation may simply be a result of the number of studies performed rather than the reflection of separate pathologies *per se*. 

## 8. GWA Studies

Advances in genetic technologies over the last decade have allowed enormous leaps in our characterization and understanding of both rare and common genetic variations at the sequence level. Projects, such as the HapMap ([91]) and 1000 Genomes ([92]), have provided us with catalogues of expected variations across multiple populations. Methodological advances, such as microarrays and high throughput genotyping and sequencing platforms, have allowed us to characterize known variants efficiently. Accordingly, gene identification shifted from linkage analysis to genome-wide association (GWA) studies [93]. GWA studies usually interrogate large cohorts of cases and controls (typical sample sizes range from 1000 up to 1,000,000), but can be extended to a regression analysis of variant frequency upon performance in phenotype-related quantitative tasks (quantitative GWA study). Since GWA studies consider the frequency of each variant independently, there is an underlying assumption that the causal variation (or a Single Nucleotide Polymorphism (SNP) in linkage disequilibrium with the causal variant) will be common across cases. As such, association studies do not allow for a high level of genetic heterogeneity between cases.

## 9. GWA Studies of Speech and Language-Related Traits

GWA studies of speech and language cohorts to date have not yielded consistent findings. Meaburn *et al.* (2008) applied a pooled genotyping method across two extreme samples selected from a twin cohort on the basis of reading ability (755 low reading ability cases and 747 high reading ability controls) using 100,000 SNPs, but did not identify any significant associations [94]. This may reflect the sparse density of the genotyping arrays employed at that time. Roeske *et al.* analyzed a discovery (N = 200) and replication (N = 186) cohort both selected for dyslexia and found association for a specific electrophysiological endophenotype of dyslexia (“mismatch negativity component” or MMN) (*p* = 5.14 × e^−8^ in combined dataset) pointing to the *SLC2A3* gene, which is implicated in glucose transport in the brain [95]. Field *et al.* 2013, performed a joint linkage and association study on 718 individuals from 101 dyslexia families with 100,000 SNPs. Again, they did not find any associations that met the threshold of genome-wide significance (1 × 10^−8^ [96]) [97]. They did however, find suggestive association (*p* = 6.2 × 10^−7^) with an SNP 77 Kb downstream of the *FGF18* gene, which has been implicated in lateralization [97]. Luciano *et al.* reported a meta-analysis of quantitative reading and language measures across two relatively large population-based samples (1177 individuals from 538 families and approximately 5000 cases) [98]. They found a suggestive level of association (*p* = 7.34 × 10^−8^) between variants in the *ABCC13* gene on chromosome 21 and non-word repetition (a marker of phonological short-term memory). However, they did not replicate the association to *FGF18* [98]. Eicher *et al.* also used a population-based sample, but in their study, they selected low language and reading performers for their GWA study [99]. Their sample included 163 language impaired probands, 353 dyslexic probands and 174 comorbid probands (*i.e.*, those with both language and reading impairment). They compared variant frequencies between these proband groups and the remainder of the population. They observed suggestive association with SNPs in *ZNF385D* (*p* = 5.45 × 10^−7^) and *COL4A2* (*p* = 7.59 × 10^−7^) in the cases with the comorbid phenotype, and to SNPs in the *NDST4* (*p* = 1.4 × 10^−7^) gene in language impaired probands [99].

Recently, it has been proposed that parent-of-origin effects could explain part of the missing heritability of complex traits, suggesting that the addition of these effects within GWA studies may be fruitful [100]. A recent study of 278 families with a language-impaired child, investigated child genotype and parent-of-origin effects [101]. They identified significant evidence for paternal parent-of-origin effects on chromosome 14q12 (*p* = 3.74 × 10^−8^) and suggestive evidence for maternal parent-of-origin effects on chromosome 5p13 (*p* = 1.16 × 10^−7^) [101]. The paternally-associated SNP on chromosome 14 yields a non-synonymous coding change within the *NOP9* gene, which has been reported to be significantly dysregulated in individuals with schizophrenia.

## 10. GWA Study Design Factors

The ability of GWA studies to identify risk variants depends upon several factors. These include the effect size of the variants, the frequency of the variants in the population under study and the population as a whole and, of course, sample size and study design [102]. The lack of replication described in the previous section is therefore perhaps not surprising: each started with a different hypothesis and definition of disorder and applied different selection procedures and association methodologies. Inconsistency across studies makes it hard to assess whether non-replication indicates the presence of false positives or is simply a function of study design or power to detect an association. In addition, the variations between independent studies means that it is not possible to simply combine existing cohorts to generate adequately powered meta-analyses.

**Figure 1:**
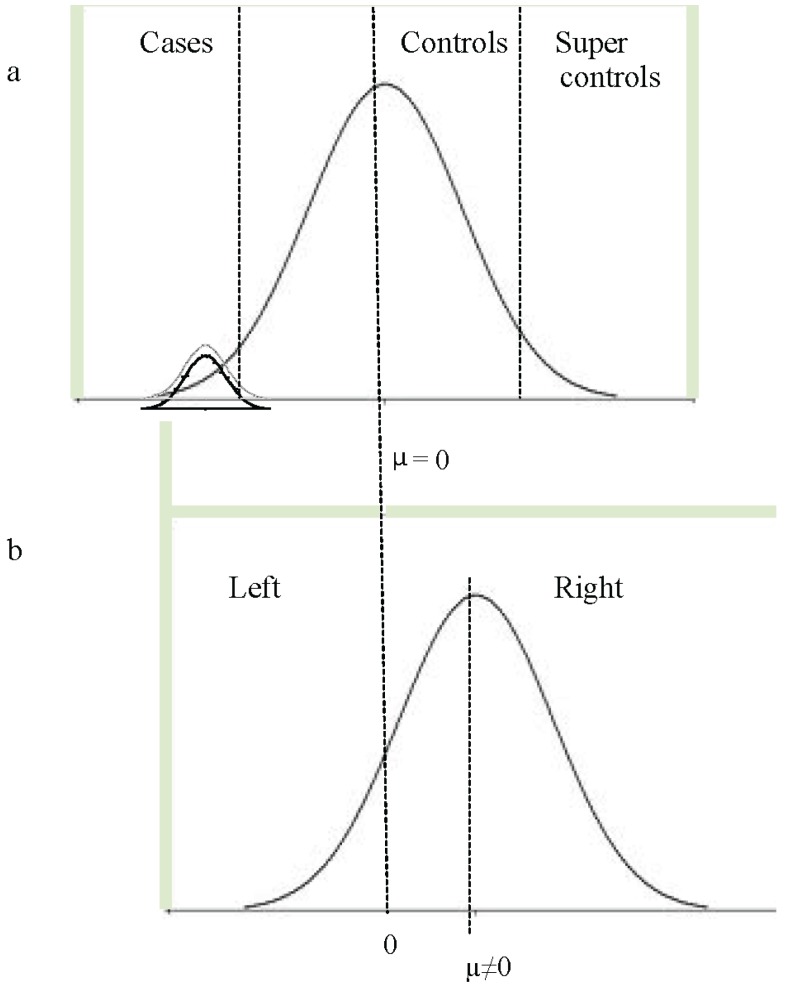
Study design for quantitative phenotypes (**a**) genome-wide association (GWA) studies for speech and language-related traits typically use phenotypes across the entire distribution (population-based quantitative GWA studies). Others might apply a binary affection status under which low language-performing individuals are defined as “cases” and individuals within the “normal” language range (usually performance above the mean) as “controls”. Under certain conditions, “super-controls” can provide more power, as they are selected to fall at the upper extreme of the distribution. If controls with phenotype data are not available, they may be derived from standard control populations under the knowledge that they might include a small proportion of cases. Quantitative GWA studies restricted to cases may be based on a phenotypic distribution restricted to the lower tail of the entire distribution or may be based on a phenotypic curve derived across cases samples, as denoted by the two normal distributions in (**a**) (note that in (**a**), the phenotype distribution may not necessarily be expected to be normal, although it is shown as such in the figure). (**b**) The pegboard test generates a quantitative measure for handedness (PegQ) that is normally distributed around a positive mean. PegQ strongly correlates with hand preference, so that individuals with positive scores are very likely to be right-handed (roughly 90% of the population), and individuals with negative scores are likely to be left handed. Typically genetic studies for handedness have used the categorical measures of hand-preference using a case-control (left *vs.* right) study design.

As discussed above, some of the language-related GWA studies used population-based samples and chose to select low language-performing subjects as cases (*i.e*., the lower tail end of the distribution, marked as “cases” Figure 1a). Others studied speech and language-related traits across the entire distribution in a quantitative GWA approach (*i.e*., the entire normal distribution in Figure 1a). Such population-based approaches assume that genetic contributions to disorders of speech and language will be the same as those that contribute to speech and language-related traits across the entire distribution. This assumption is dependent upon the way in which disorders, such as SLI and dyslexia, are conceptualized: do they just represent the lower tail of the normal distribution with respect to speech and language (dimensional model) or is there a qualitative difference between dyslexic individuals and poor readers (categorical model)? (*i.e*., the difference between the “cases” and the lower normal distribution in Figure 1a).

## 11. Dimensional and Categorical Models of Language Disorder

The distinction between dimensional and categorical models of language disorders is still a matter of debate. Leonard argues that perhaps there is no tangible cause for SLI, and the mental representations of children with the label SLI are not distinct from other children [103]. Taxonomic and principal component analyses support this view, demonstrating that relationships between language-related measures do not differ between children affected by SLI and those with normal language development [104,105,106]. In addition, the ease of the acquisition of specific language features has also been shown to be consistent between children affected by SLI and those with normal language development [107]. Under this dimensional model, one can consider variation across the range when attempting to identify underlying genetic effects. Twin studies of SLI instead support a categorical distinction [4,108,109,110,111]. Such studies indicate that children who have speech and language difficulties that are severe enough to warrant clinical referral have a qualitatively different profile of impairment, which shows increased levels of heritability [110]. Thus, it is possible that some forms of speech and language impairment, at least, have different underlying pathology from those mechanisms that are important in normal language variation (*i.e*., the distinct lower normal distribution in Figure 1a). Under this hypothesis, one needs to specifically select children with SLI to identify these distinct underlying genetic effects. It is, of course, possible that both models contribute to some level: studies of the effects of specific genetic risk variants upon language development indicate that some genetic risk variants play a role across the entire distribution of ability [87,112,113,114], while others appear to play a role that is specific to impairment [87,113]. These data suggest that a variety of approaches will be required to delineate genetic effects underlying language impairment: GWA studies of individuals across a range of abilities will identify genes that contribute across the entire distribution, while studies of specific disorder subsets will be required to identify genes with more specific effects. 

## 12. Cross-Linguistic Difficulties of Speech and Language Disorders

Given all these complexities, we extol the value of combining quantity with quality in the collection of larger cohorts carefully characterized at the phenotypic level. This is a major investment both in time and resources, but clearly represents the most promising way forward. The success of genetic mapping for complex traits has been largely facilitated by the collaboration of scientists and clinicians in large consortia, which have facilitated the collection of large sample sizes for genome mapping. This normally requires international collaborations, which pose additional complications for reading and language disorders. The psychometric tests used to assess reading and language skills cannot be separated from the language spoken in different countries and are not always directly comparable. In this context, the European NeuroDys project is working to define common guidelines for the collection and assessment of a large dyslexia cohort throughout research groups spread across Europe [115]. NeuroDys aims to exclude comorbidity and select severe dyslexic cases by selecting those more than 1.25 SD below grade level on a standardized word-reading test. Their strategy also includes the screening of control samples. Since dyslexia has a relatively high frequency, 5%–10% of population samples, routinely employed in GWA studies, will be expected to consist of individuals with dyslexia. GWA analysis of the NeuroDys cohort is currently underway. Analysis of dyslexia candidate genes in this cohort did not reveal any statistically significant association, highlighting many of the challenges covered in this review [116]. As discussed previously, universal inclusion/exclusion criteria are a good strategy to facilitate meta-analyses or cross-linguistic categorical GWA studies. Nonetheless, they do not entirely solve the challenge of obtaining large sample sizes for quantitative analyses. For example, single word reading tests tend to assess accuracy in English *versus* speed in transparent languages, like Italian or German. A composite score, including both accuracy and speed, as used by the NeuroDys study, can address this, but does not necessarily reflect the real reading difficulty in different populations. It is interesting to note that despite these differences, associations with candidate genes have been replicated in different languages; association with the *DCDC2* gene has been reported in English-speaking (Meng *et al.* 2005 [61], Scerri *et al.* 2011 [113]) and German-speaking (Schumacher *et al.* 2006 [117]) cohorts. Similarly, cross-linguistic studies find that features of the native language can act to modulate given aspects of the SLI phenotype. In general, those linguistic features that are considered “hardest” for a normally developing child to understand will represent areas of particular problem for children with SLI [118,119]. Within-child differences indicate that this generalization extends to bilingual children. Bilingual children with SLI encounter similar problems to monolingual children with SLI, and these problems appear to be language-specific [120]. These cross-linguistic variations represent an extra complication for meta-analyses and collaborative studies of speech and language impairments. If the features of the disorder vary across populations, the measurement of disability will be language-specific. Thus, multi-site efforts will not only need to consider the best way to overcome cross-ethnicity genetic differences, but also cross-ethnicity language differences.

## 13. Mega-, Meta- and Mixed-GWA Studies

Many successful GWA studies in the literature now include hundreds of thousands of individuals (diabetes, body mass index, height) [121,122,123,124,125] compared to the hundreds employed in the studies described above. Such “mega-GWA studies” have been relatively successful in identifying significant and consistent loci that contribute to either disease status or continuous traits across the general population. However, it is clear from these large studies that each genetic risk variant is likely to explain only a small amount of the heritability and that several hundred risk loci are likely to underlie any given trait or disorder [126]. Besides an increased sample sizes, a possible route of future investigation may be provided by a model adopted in the study of psychiatric disorders. Much like SLI and dyslexia, there is much evidence for the existence of shared genetic effects between psychiatric disorders, such as bipolar disorder, depressive disorder and schizophrenia. “Mixed-GWA study” investigations of mixed cohorts across these disorders have recently identified risk factors that span these clinical boundaries, suggesting that there may be some common pathophysiologies across related disorders [127,128]. The above studies suggest that meta-analyses across the existing SLI and dyslexia cohorts would be a worthwhile effort, despite the challenges involved in the amalgamation of these highly heterogeneous samples.

## 14. Quantitative *vs.* Qualitative, or Both

Throughout this manuscript, we have clearly advocated the combined analyses of existing cohorts or the collection of new large homogeneous samples relevant to developmental disorders. However, is sample size all that matters? If that were the case, the field should probably shift towards questionnaire-based phenotypic assessments, which would be an efficient way to boost numbers. Nonetheless, there is an intrinsic value in investing in phenotypic assessments that are detailed and quantitative. Firstly, this strategy allows cohorts to be stratified in distinct subgroups according to distinct criteria, which might be required for different hypotheses. For example, it makes it possible to select for the severity of disorders or to change features along with revised diagnostic criteria (as in Figure 1b). In addition, association analyses that use quantitative measures are potentially more powerful, as they exploit the full range of variability of a given phenotype, allowing the direct investigation of dimensional and categorical models. A recent story describing a GWA study for handedness is emblematic of how small, detailed cohorts can provide power to detect relevant biological effects [129,130]. This GWA study was conducted in a small sample (N = 728, well below the recommended standard) originally selected for a dyslexia diagnosis. In addition to language-related measures, this cohort was characterized with the pegboard test, which allows the derivation of a quantitative measure of relative hand skills (PegQ). This measure is normally distributed with a right-shifted mean and strongly correlates with hand preference (Figure 1b). Despite the small sample size, the screening led to a genome-wide significant result within the *PCSK6* gene, known to regulate the NODAL protein pathway, essential for the left/right patterning in early embryonic development [131], and therefore represents an extremely interesting candidate for a lateralized phenotype. Although this association was replicated in two independent cohorts with developmental dyslexia, the risk variant appeared to confer an opposite effect (reduced relative laterality) in a general population cohort (N = 2666). In support of these findings, subsequent gene enrichment analysis showed that genes controlling structural asymmetries were associated with the handedness measure in both cohorts regardless of a dyslexia diagnosis [130]. These findings demonstrate the importance of studying phenotypes within specific selected cohorts, as well as across the general population, as discussed above. It is possible that these effects are the result of risk variant interactions with different genotypic/biological backgrounds, as discussed below. A dyslexia-specific effect has also been observed for other traits. A variant in the *MYO18B* gene has also been implicated in mathematical ability specifically in children with dyslexia [132]. Hand preference (left *vs.* right) can readily be collected as a simple add-on question in any questionnaire battery. Thus, it would be very straightforward to re-analyze or meta-analyze existing GWA cohorts (even those collected for the investigation of different traits) using a case-control definition based on hand preference. A recent conference abstract describing a genome-wide analysis of hand preference data from more than 20,000 individuals did not find any genome-wide significant loci (Medland *et al.* ASHG, 2009 [133]). Similarly, a study of 4268 subjects from a population-based cohort, which included broad measures of laterality (hand or foot preference, ocular dominance or hand clasp), did not yield genome-wide significant associations [134]. These data suggest that the genetic effects at the population level are extremely low or, alternatively, that a categorical approach is not suitable for dissecting the genetics of this trait. It is likely that the biological regulation of hand preference involves complex and integrated processes that are not efficiently represented by the reductive phenotypes of left- *versus* right-handedness. If this can be said for a relatively transparent trait, like handedness, then we might expect these correlations to be even lower when considering a phenotype as complex as language. In addition, we must consider that the genetic effects might depend on sample stratification for a neurodevelopmental disorder definition. 

## 15. Filling the Gap

Collecting large-scale GWA cohorts takes time, effort, funding and usually a concerted and collaborative effort between multiple research and clinical teams. Does this mean that we cannot make progress in this field until such statistical criteria are met? Perhaps the application of next-generation sequencing technologies to existing sample sets with detailed phenotypic information has the potential to fill this gap. While small individual family-based units do not provide enough power to map variants through linkage analyses alone, the application of this technique in combination with high-throughput sequencing can provide a powerful paradigm. The whole-exome or whole-genome sequencing of sporadic cases and their parents under the assumption of causative *de novo* mutations has proven successful in disorders, such as autism [135,136,137,138] and intellectual disability [139]. The sequencing of larger family units with multiple recurrent cases have allowed the identification of “ultra-rare” (often defined as <0.01%) or private (unique to the given family) mutations with high functional impact (*i.e*., the gain of a stop codon and frame shift mutations) that cosegregate with disorder [140,141]. A recent exome sequencing study of children with childhood apraxia of speech investigated known candidate genes for language development [142]. They reported potentially deleterious variants in *FOXP1*, *CNTNAP2*, *ATP13A4*, *CNTNAP1*, *KIAA0319* and *SETX*, providing support for the role of these candidate genes [142].

## 16. Difficulties of High-Throughput Sequencing Studies

Although relatively simplistic in design, high throughput sequencing studies are far from straightforward. Rigorous quality control procedures and expert bioinformatics are needed, and the variation between platforms, capture assays and algorithms can be problematic. The main problem, though, is perhaps the proof of causality. The number of *de novo* mutations can be affected by environmental factors (e.g., paternal age [143]), but all being equal, the expected number of *de novo* mutations is approximately one per exome per generation. In contrast, the number of private mutations identified in an exome can be substantial, particularly as sequencing sensitivity and coverage increase. In addition, by design, these methods focus upon mutations that are likely to be private to the given individual and will not generalize between families. Studies of autistic disorder indicate that recurrent mutations in specific genes will be rare [135,136,137]. Furthermore, while the role of rare disruptive mutations is perhaps more tangible than that of common variation, these effects are still likely to function as part of a complex genetic model and represent risk variants rather than a causative mutation. Incomplete segregation, even within family units, is often observed for both rare mutations and copy number events, which can also prove a fruitful avenue of research in relatively small sample sizes. While large, disruptive events of genes that are known to be important can clearly be assigned some functionality, when a mutation or copy number variant is private, it can be very hard to distinguish between a functional effect that is subject to incomplete penetrance or modifier gene effects and a non-functional change. Such observations have led to the double-hit hypothesis in developmental disorders in which mutations and/or copy number events combine in an additive or epistatic manner to modulate the exact clinical presentation [136,144,145,146,147,148].

## 17. Translational Relevance

The pathway from association to functional evidence is a long, but necessary road if we are to truly elucidate the biological mechanisms underlying speech and language disorders. While a *p*-value < 0.5 × 10^−8^ or the observation or private mutations in conserved motifs are certainly robust indicators of a genuine genetic susceptibility, these findings remain statistical predictions if they are not coupled by functional data. It is important to keep in mind that the risk variants identified by GWA studies are not necessarily functional and often represent proxies for the functional variant [102]. Functional investigations are being further progressed by projects, such as the Encyclopedia Of DNA Elements (ENCODE) and the Genetic European Variation in Health and Disease (GEUVADIS) project, which apply high-throughput methods to the study of gene expression and regulation to facilitate our understanding of the findings of GWA studies [149,150]. The functionality of rare coding mutations or copy number events is more tractable, but still not straightforward, especially when they are private. Changes to the coding sequence do not always result in protein dysfunction, and the interpretation of the severity of a given mutation often relies on *in silico* predictions [151]. Once the identity of the functional variant is established, its biological contribution to disorder needs to be clarified, a step which is usually achieved in animal models. The development of translational targets is not always clear, even for fully penetrant monogenic mutations with clear functional effects [152,153]. These limitations reflect the importance of considering genetic variants within relevant pathways and networks and are likely to be exacerbated for complex disorders.

## 18. Biology beyond the *p*-Values

The association of variants in the *PCSK6* gene and relative hand skill introduced earlier acts to illustrate the importance of taking statistical associations forward with molecular investigations of the mechanism. As described above, the *PCSK6* association with relative hand skill appears to confer specific effects in individuals with dyslexia. Given that the prevalence of left-handers is not higher among individuals with dyslexia compared to controls, at first glance, it appears difficult to explain this specificity. The very fact that this handedness measure was collected in individuals with dyslexia stems from a long-sought link between laterality and neurodevelopmental disorders [154]. Language is a lateralized behavior under the control of one specialized cerebral hemisphere (the left one, in most cases), and it has been suggested that language-related disorders might therefore be linked to handedness, which is the most obvious lateralized behavior. For many decades, researchers have looked with little success for a link between handedness and different psychiatric disorders, mainly by assessing the frequency of left-handers in patient cohorts [155]. Whilst largely inconclusive, an increased frequency of left-handers has been reported for schizophrenia [156]. Furthermore, imaging studies have shown that disorders, such as dyslexia and SLI, present with atypical and weaker cerebral lateralization [25]. Although, there is some controversy over whether these effects are causative or consequential [157].

## 19. Converging Evidence towards Biological Pathways

More direct evidence in support of the dyslexia-specific *PCSK6* association comes from very recent functional studies. We have already eluded to the apparently connected functional role of dyslexia candidate genes. Functional studies and *in vivo* techniques have demonstrated that several of the dyslexia candidates described above (*KIAA0319*, *DCDC2* and *DYX1C1*) are involved in early phases of fetal brain development and, in particular, in neuronal migration processes. In addition, *ROBO1* is a neuronal axon guidance receptor that is also important for cortex development. The importance of neuronal migration processes in dyslexia is thus now generally accepted in the literature [158]. However, it is not clear which exact mechanisms these candidate genes mediate. More recently, a role for cilia function and development has also been suggested as a common biological pathway between dyslexia candidate genes [159]. Cilia are essential in the establishment of left/right axis determination in the first weeks of embryogenesis by the activation of the NODAL pathway on the left side of the embryo. Mutations in genes controlling cilia function lead to a class of conditions often characterized by laterality defects (ciliopathies). *DCDC2*, *DYX1C1* and *KIAA0319* have recently been reported to form a novel co-expressed module in ciliated cells [79]. *Dcdc2* has been implicated in the control of cilia length [72], and disruption of *Dyx1c1* in mice and zebrafish [77,78] leads to laterality defects and impairments in cilia function, resembling that observed in primary ciliary dyskinesia (PCD). In humans, *DYX1C1* mutations have been identified in 12 families with PCD [77]. *KIAA0319* is characterized by five polycystic kidney disease (PKD) domains [160] typically found in proteins (e.g., *PKD1* and *PKD2*) playing key roles in cilia function [161].

Cilial structures control many processes. In particular, they are important in neuronal migration, where they play a guiding role during cortical development. It has therefore been suggested that the roles of cilia in neuronal migration may be directly implicated in leading to cortical defects, which are at the basis of cognitive deficits in neurodevelopmental disorders [162]. Taken together, these observations support a possible interaction between biological pathways controlling the establishment of left/right structural asymmetries and neuronal migration early in development with genes implicated in dyslexia [159]. These interactions could be mediated by concomitant actions in controlling cilia function and could explain the *PCSK6* association observed specifically in individuals with dyslexia. Furthermore, these data suggest for the first time that the mechanisms that control left/right body asymmetries maybe also be relevant in establishing functional brain asymmetries, contrary to previous evidence [163]. Incorrect reference order

## 20. Conclusions

We have discussed the various factors that can impact upon the relative success of genetic investigations in general and highlighted those factors that may be particularly pertinent to the investigation of speech and language disorders. While GWA studies have often been criticized for their high economic cost and little clinical benefits, they have contributed enormously to our understanding of human genome variation and appreciation of the complexity behind human biology and genetic disorders. Nonetheless, the application of these technologies to the study of genetic contributions to language and reading has not progressed as fast as other complex traits. One obvious limitation to the study of speech and language is the challenge of defining cases and ascertaining homogeneous cohorts with phenotypic measures that are universally relevant at the biological level. The translational application of genetic research to complex disorders will require the integration of large genomic datasets, functional genomic screenings and basic research projects aimed at studying the human brain. We know that genes and proteins do not act in isolation, and their functions differ between individuals, tissues, environments and over time; yet, we still consider risk variants as independent entities with fixed effects. Although we must accept that it is not currently possible to simultaneously consider all these effects within a single model, perhaps it is time to question the adherence to clinically defined strata in the genetic study of developmental disorders and, instead, promote the acceptance of cross-disorder studies that fully consider comorbidities across clinical symptoms and environmental factors, as well as regulatory effects and epigenetics. This reiterates the need for multidisciplinary collaborations to enable an increase in sample sizes, while maintaining detailed phenotypic assessments that ultimately will inform the definition of diagnostic criteria. In terms of future directions, it is difficult to establish whether resources would be better spent collecting large cohorts that meet a superficial categorical cutoff or, alternatively, in assembling smaller cohorts characterized across a range of detailed developmental phenotypes. What is clear is that the definition of universal guidelines will facilitate the coordinated collection of uniform and international cohorts, easing the burden of downstream analyses. Although larger collaborations are needed, a meta-analysis of existing resources would be a good starting point to establish such efforts. The reporting of complete data sets, even when events are considered non-significant or uninteresting in isolation, will also be crucial to the planning of future collections. Ultimately, functional evidence is necessary to definitely prove the downstream effects of genetic variants and to understand the biology of underlying disorders and neurodevelopment. Despite the lack of advances from genome-wide screening in speech and language disorders, the functional assessment of candidate genes has allowed considerable progress in the identification of specific biological processes that may be important in these phenotypes. In particular, neuronal migration and ciliogenesis have been highlighted as two, perhaps related, processes that may play a role in developmental dyslexia. These encouraging findings demonstrate the importance of the systematic integration of functional studies and genetic association or sequencing studies. 
